# Analysis of inflammation- and atherosclerosis-related gene polymorphisms in branch retinal vein occlusion

**Published:** 2009-03-27

**Authors:** Iris Steinbrugger, Anton Haas, Richard Maier, Wilfried Renner, Monika Mayer, Christoph Werner, Andreas Wedrich, Yosuf El-Shabrawi, Otto Schmut, Martin Weger

**Affiliations:** 1 Department of Ophthalmology, Medical University of Graz, Graz, Austria; 2 Clinical Institute of Medical and Chemical Laboratory Diagnostics, Medical University of Graz, Graz, Austria

## Abstract

**Purpose:**

Branch retinal vein occlusion (BRVO) is a common vision-threatening disease. Compression of the underlying retinal vein due to increased rigidity of the crossing artery has been implicated in the pathogenesis of BRVO. Among others, arterial hypertension and hypercholesterolemia, both of which contribute to atherogenesis, have been identified as risk factors. Atherosclerosis itself is a chronic low-grade inflammatory disease with a distinct pro-inflammatory cytokine pattern. In addition to their role in atherogenesis, some cytokines have been shown to exert procoagulatory effects, and may thus contribute to the development of BRVO by a second mechanism. Gene polymorphisms affecting the expression of inflammation-related cytokines are therefore candidates as potential risk factors for BRVO. The purpose of the present study was to investigate hypothesized associations between cytokine gene polymorphisms and the presence of BRVO.

**Methods:**

The study comprised 398 patients with BRVO and 355 control subjects. Using 5′exonuclease assays (TaqMan), genotypes of the following functional single nucleotide polymorphisms were determined: interleukin 1 beta (*IL1B*) −511C>T, interleukin 1 receptor antagonist (*IL1RN*) 1018T>C, interleukin 4 (*IL4*) −584C>T, interleukin 6 (*IL6*) −174G>C, interleukin 8 (*IL8*) −251A>T, interleukin 10 (*IL10*) −592C>A, interleukin 18 (*IL18*) 183A>G, tumor necrosis factor (*TNF*) −308G>A, monocyte chemoattractant protein 1 (*CCL2*) −2518A>G, and RANTES (*CCL5*) −403G>A.

**Results:**

Neither genotype distributions nor allele frequencies of any of the investigated polymorphisms differed significantly between BRVO patients and controls (p>0.05). Arterial hypertension was found to be significantly more prevalent in BRVO patients than in controls (p<0.001). In a logistic regression analysis presence of arterial hypertension was associated with an odds ratio of 3.33 (95% confidence interval: 2.42–4.57) for BRVO.

**Conclusions:**

As none of the investigated gene variants was significantly more prevalent in BRVO patients than among control subjects, our data suggest that these polymorphisms themselves are unlikely major risk factors for BRVO.

## Introduction

Branch retinal vein occlusion (BRVO) is a vision-threatening disease, commonly affecting patients older than 60 years. It frequently occurs at an arteriovenous crossing site, where the retinal vein shares a common adventitial sheath with the retinal artery [1,2]. Increased rigidity of the crossing artery resulting from atherosclerotic disease has been suggested to cause compression of the underlying vein, resulting in turbulent blood flow, endothelial damage, and thrombus formation [3]. Consequently, arterial hypertension, elevated plasma homocysteine concentrations, and hypercholesterolemia, all of which are known to contribute to atherogenesis, have been identified as BRVO risk factors [4-7].

Atherosclerosis itself is a chronic low-grade inflammatory disease, which is characterized by the recruitment of both monocytes and T lymphocytes to the site of inflammation and a distinct pro-inflammatory T_H_1 cytokine pattern [8,9]. Animal experiments have provided some evidence that a lack of anti-inflammatory cytokines promotes atherogenesis, whereas deficiency of pro-inflammatory cytokines is associated with reduced atherosclerosis [10-12]. Importantly, cytokines and chemokines such as interleukin 1 beta (IL-1β), interleukin 6 (IL-6), interleukin 8 (IL-8), tumor necrosis factor alpha (TNF-α), and monocyte chemoattractant protein 1 (MCP-1) have also been reported to affect hemostasis, and may thus contribute to thrombus formation [13-17].

Single nucleotide polymorphisms (SNPs) affecting the expression of cytokines or chemokines previously implicated in atherogenesis as well as activation of the coagulation cascade are thus potential risk factors for BRVO. Nevertheless, they have not yet been investigated among BRVO patients. Consequently, in the present study, functional gene polymorphisms encoding the following cytokines or chemokines were chosen as candidate risk factors.

The pro-inflammatory cytokine IL-1β exerts strong pro-atherogenic effects by enhancing expression of intercellular adhesion molecule 1 (ICAM-1) and vascular cell adhesion molecule 1 (VCAM-1) on endothelial cells. Furthermore, IL-1β induces the synthesis of chemokines, such as IL-8 and MCP-1, and thus facilitates the transendothelial migration of inflammatory cells [18,19]. Additional evidence for its role in atherosclerosis comes from an animal model showing that in apoE-deficient mice a lack of IL-1β is associated with decreased severity of atherosclerosis [20]. IL-1β also exerts procoagulatory effects by increasing the expression of tissue factor, which itself plays an essential role in the activation of the extrinsic pathway of the coagulation cascade [13]. Furthermore, increased expression of IL-1β downregulates tissue–type plasminogen activator (t-PA), and thus impairs fibrinolysis [21]. A gene polymorphism, characterized by a C to T transition at position –511 of the *IL1B* gene (*IL1B*−511C>T, rs16944), affects the expression of *IL1B*. Iacoviello and coworkers [22] found that stimulation with lipopolysaccharide (LPS) produced a significant decrease in tissue factor expression as well as IL-1β release in mononuclear cells from participants carrying the −511TT genotype compared to those homozygous for the −511C allele.

By binding to the IL-1 receptor without transmitting the agonist signal, the interleukin 1 receptor antagonist (IL-1Ra) counter-regulates IL-1β activity [18]. Animal models have demonstrated that deficiency of IL-1Ra affects the development of atherosclerotic lesions [23,24]. Recently, a polymorphism within the gene encoding IL-1Ra (*IL1RN* 1018T>C, rs4251961) has been associated with both decreased IL-1Ra production and increased IL-6 and C-reactive protein (CRP) plasma concentrations [25].

Several studies support an important pathogenic role of interleukin 4 (IL-4) in atherogenesis. IL-4 mediates proatherogenic pathways by both increasing the expression of MCP-1, IL-6, VCAM-1, and by upregulation of 15–lipoxygenase, which itself plays an essential role in oxidizing low-density lipoprotein (LDL) to its atherogenic form [26-29]. Furthermore, mice experiments have provided evidence that IL-4 deficiency is associated with decreased formation of atherosclerotic lesions [30,31]. IL-4 promotes fibrinolysis by stimulating monocytes to produce t-PA [32]. A functional IL-4 polymorphism, which is characterized by a C to T substitution in the promoter region of the *IL4* gene (*IL4* −584C>T, rs2243250), has been identified; thus far, it not been studied among BRVO patients [33].

IL-6 is a pleiotropic, pro-inflammatory cytokine, which is synthesized by monocytes and vascular endothelial cells [34]. It has been shown to contribute to atherogenesis by induction of endothelial dysfunction and enhancement of the expression of adhesion molecules [35]. In animal experiments, IL-6 has been observed to promote the development of early atherosclerotic lesions, thus strongly confirming an essential role of IL-6 in atherogenesis [10]. Furthermore, IL-6 promotes coagulation by upregulating the transcription of tissue factor, fibrinogen, and factor VIII [36-38]. Interestingly, IL-6 plasma levels have been reported as a risk determinant for venous thrombosis [39]. In 1998, Fishman and coworkers identified a functional gene polymorphism in the promoter region of the *IL6* gene, which is characterized by a C to T substitution at position −174 (*IL6*−174G>C, rs1800795). Using transfected HeLa cells, Fishman and colleagues found higher baseline IL-6 levels in cells with the G construct compared to those transfected with the C allele. Stimulation with LPS or IL-1 resulted in a significantly increased IL-6 transcription rate among cells carrying the G allele [40]. Another study using anti CD3/CD28-stimulated peripheral blood lymphocytes found three times higher IL-6 concentrations among carriers of the G allele [41]. Interestingly, the *IL6*−174G>C polymorphism has previously been suggested to affect susceptibility to retinal artery occlusion (RAO). Its role in BRVO, however, still remains elusive [42].

In contrast to IL-6, interleukin 10 (IL-10) is an anti-inflammatory T_H_2-associated cytokine that mediates anti-atherogenic pathways by suppressing the synthesis of several pro-inflammatory cytokines such as IL-1β, IL-6, interleukin 12 (IL-12), TFN-α, and interferon-gamma (IFN-γ) [43]. Its protective role in atherogenesis has also been confirmed by animal experiments [12]. As for hemostasis, IL-10 has been shown to inhibit LPS induced tissue factor expression in monocytes [44]. Gene polymorphisms are thought to account for up to 75% of the variability of IL-10 production [45], and increased IL-10 expression has been associated with the presence of a TCATA haplotype formed by polymorphisms at position −3575, −2763, −1082, −819, and −592 in the promoter region of the *IL10* gene [46,47]. Due to strong linkage disequilibrium, the presence of this haplotype can be fully determined by analysis of the *IL10*−592C>A polymorphism (rs1800872). The *IL10*−592A allele indicates the presence of the presumed anti-inflammatory TCATA haplotype, while the −592C allele indicates its absence. As for retinal vascular diseases, an association between the *IL10*−592C>A polymorphism and RAO risk has been reported [48].

Animal models have also provided evidence that the pro-inflammatory cytokine interleukin 18 (IL-18) contributes to the development of atherosclerosis. In synergy with IL-12, IL-18 promotes the T_H_1 immune response by inducing the IFN-γ synthesis [19]. It also upregulates the synthesis of pro-inflammatory cytokines such as IL-1β and IL-8 as well as the expression of adhesion molecules [49]. In apoE knockout mice, administration of IL-18 has been associated with enhancement of atherosclerosis, whereas deficiency of IL-18 has been shown to reduce the extent of atherosclerosis [50,51]. IL-18 expression has been found to be decreased among carriers of the GCAGT haplotype formed by polymorphisms at position −887, −105, +105, +183, and +533 in the promoter and 5′untranslated region of the *IL18* gene [52]. By analysis of the *IL18*+183A>G polymorphism (rs5744292) the GCAGT haplotype can be identified, since this haplotype is the only one including the +183G allele. Thus, the *IL18*+183G allele indicates the presence of this haplotype, while the +183A allele indicates its absence.

Overexpression of TNF-α, a potent pro-inflammatory cytokine mainly produced by macrophages and T cells, has also been implicated in the pathogenesis of atherosclerosis [19]. TNF-α upregulates both the expression of adhesion molecules (ICAM-1, VCAM-1) and chemokines such as MCP-1, thereby facilitating the recruitment of monocytes to atherosclerotic lesions [53]. In apoE-deficient mice a lack of TNF-α was observed to be associated with a reduced size of atherosclerotic lesions [54]. TNF-α also exerts procoagulatory effects by upregulating tissue factor expression and by inhibition of the thrombomodulin/protein C anticoagulation pathway [14,15]. In addition, impairment of fibrinolysis is caused by TNF-α induced t-PA downregulation [55].

A *TNF* promoter polymorphism, which is caused by a G to A substitution at position −308 of the *TNF* gene (*TNF*-308G>A, rs1800629), has been reported to modulate the expression of TNF-α. The presence of the *TNF*-308A allele has been found to be associated with higher constitutive and inducible TNF-α levels after LPS stimulation compared to the −308GG genotype [56].

Importantly, the migration of leukocytes to the site of inflammation occurs along a gradient of various chemokines including MCP-1, IL-8, and RANTES (regulated on activation, normal T cell expressed and secreted). MCP-1 belongs to the CC family of chemokines, and its expression in both endothelial cells and monocytes can be induced by pro-inflammatory cytokines such as IL-1, IFN-γ, and TNF-α [57]. A large body of evidence indicates that MCP-1 plays an essential role in atherogenesis by recruiting monocytes and macrophages to atherosclerotic lesions [58,59]. MCP-1 has also been reported to have a procoagulant function by inducing tissue factor expression [60]. An A to G substitution at position −2518 of the MCP-1 gene (*CCL2*−2518A>G, rs1024611) has been noted to affect MCP-1 expression [61]. After IL-1β stimulation, peripheral blood mononuclear cells from individuals carrying the −2518G allele showed a significantly higher MCP-1 synthesis than cells from individuals homozygous for the −2518A allele [61].

Endothelial secretion of IL-8, a member of the CXC chemokine family, is induced by pro-inflammatory cytokines such as IL-1 and TNF-α [62]. IL-8 is a potent chemoattractant for neutrophils, and, like MCP-1, also triggers firm adhesion of rolling monocytes to the vascular endothelium, thus contributing to the development of atherosclerotic lesions [63]. Decreased susceptibility to atherosclerosis has been demonstrated in mice lacking the IL-8 receptor [64]. IL-8 has also been shown to increase the expression of tissue factor in monocytes [13]. Hull and coworkers [65] identified a common SNP in the promoter region of the *IL8* gene, which is caused by an A to T substitution at position −251 (*IL8*−251A>T, rs4073). The *IL8*−251T allele was found to be associated with a two- to fivefold stronger luciferase expression than the −251A allele, suggesting a pro-inflammatory effect in carriers of this allele [66].

Finally, RANTES, a member of the CC family of chemokines, is produced by T lymphocytes and monocytes. As a potent chemoattractant, RANTES has been shown to trigger monocyte arrest on dysfunctional endothelium [67]. Furthermore, RANTES is expressed by T cells in advanced atherosclerotic lesions [68]. In a hypercholesterolemic mouse model, administration of the CC chemokine antagonist Met-RANTES resulted in reduced atherosclerotic lesion sizes [69]. A SNP in the promoter region of the RANTES gene (*CCL5*), which is caused by a G to A substitution at position −403 (*CCL5*−403G>A, rs2107538) has been identified [70]. In cells transfected with the −403A variant, increased transcriptional activity has been demonstrated.

So far none of these functional gene polymorphisms has been studied as a potential risk factor for BRVO. The purpose of the present study was thus to investigate a hypothesized association between the aforementioned gene polymorphisms and the presence of BRVO.

## Methods

The present study enrolled 398 patients with BRVO (228 females and 170 males) and 355 control subjects (206 females and 149 males). The mean age of patients was 67.1±11.0 years (range: 37–93 years) and 68.3±13.8 years (range: 21–93 years) in the control group. All participants were of Caucasian origin, living in the same geographical area and were seen at the local Department of Ophthalmology, Medical University of Graz. The study was approved by the Institutional Review Board of the Medical University of Graz and followed the principles of the Declaration of Helsinki. Prior to enrollment, written informed consent was obtained from all participants. Diagnosis of BRVO was made if fundus examination revealed venous dilation and tortuosity with intraretinal hemorrhages in a wedge-shaped region, with the apex of the wedge pointing to an arteriovenous crossing point.

The control group was hospital-based and comprised patients seen at the Department of Ophthalmology, Medical University of Graz for reasons other than retinal vascular occlusion, such as cataract or glaucoma surgery. All controls underwent a detailed fundus examination. Excluded as controls were patients who had a history of retinal vascular occlusions, deep vein thrombosis, pulmonary embolism, myocardial infarction, or stroke.

Arterial hypertension was defined by a systolic blood pressure ≥140 mmHg, a diastolic blood pressure of ≥90 mmHg, or the intake of antihypertensive drugs. Participants were classified as diabetics if they were being treated for insulin or non-insulin-dependent diabetes mellitus. Hypercholesterolemia was defined by fasting plasma cholesterol levels above 200 mg/dl or the intake of lipid-lowering drugs. Participants were classified as either ever (current/previous) smokers or nonsmokers. A standardized questionnaire was employed to obtain data on medication intake as well as history of deep vein thrombosis, pulmonary embolism, myocardial infarction, and stroke.

Candidate gene polymorphisms were selected using the following criteria: association of the polymorphism with gene expression or activity of the gene product; involvement of the gene product in pro- or anti-inflammatory pathways; a minor allele frequency of 5% or higher; and no data available on the role of this gene variant in BRVO. Genomic DNA was isolated from whole blood by standard methods (QIA-AMP DNA, blood mini kit, Qiagen, Vienna, Austria) and stored at −20 °C. Genotypes of the aforedescribed polymorphisms were determined by 5′ exonuclease assays (TaqMan). Applied Biosystems Assay-by-Design custom service (Applied Biosystems, Vienna, Austria) was used for design and manufacture of primer and probe sets. Sequences of primers and probes are presented in Table 1. Endpoint fluorescence data were exported into Excel format and analyzed as scatter plots. Samples were analyzed in batches, each containing 94 samples and 2 negative controls (water instead of DNA). Next, 50 samples were reanalyzed, and the results were identical for all samples.

**Table 1:** Sequences of primers and probes for TaqMan genotyping assays.

**Polymorphism**	**Primer/probe**	**Sequence**
*IL1B -*511C>T (rs16944)	forward primer	GAGGCTCCTGCAATTGACAGA
	reverse primer	TCTCTACCTTGGGTGCTGTTCT
	C probe	VIC-CTGCCTCGGGAGCT-NFQ
	T probe	FAM-CTGCCTCAGGAGCT-NFQ
*IL1RN* 1018T>C (rs4251961)	forward primer	CCGGTGAGCCCTAAGTCTAAGATAG
	reverse primer	GCCCTTCAGACCTCATTTTGACA
	T probe	VIC-AAAATGGACCTGATGCTAT-NFQ
	C probe	FAM-AATGGACCTGGTGCTAT-NFQ
*IL4* -584C>T (rs2243350)	forward primer	GACCTGTCCTTCTCAAAACACCTAA
	reverse primer	GGCAGAATAACAGGCAGACTCT
	C probe	VIC-CATTGTCCCCCAGTGCT-NFQ
	T probe	FAM-CATTGTTCCCCAGTGCT-NFQ
*IL6* -174G>C (rs1800795)	forward primer	GACGACCTAAGCTGCACTTTTC
	reverse primer	GGGCTGATTGGAAACCTTATTAAGATTG
	G probe	VIC-CCTTTAGCATCGCAAGAC-NFQ
	C probe	FAM-CTTTAGCATGGCAAGAC-NFQ
*IL10* -592C>A (rs1800872)	forward primer	GGTAAAGGAGCCTGGAACACATC
	reverse primer	GCCCTTCCATTTTACTTTCCAGAGA
	C probe	VIC-CCCGCCTGTCCTGTAG-NFQ
	A probe	FAM-CCGCCTGTACTGTAG-NFQ
*IL18* +183A>G (rs5744292)	forward primer	AGCTGAGTGTAGTGACGCATG
	reverse primer	CTCCTGCCTCAGCCTCTTG
	A probe	VIC-CCTCAATCCCAGCTACT-NFQ
	G probe	FAM-CTCAATCCCGGCTACT-NFQ
*TNF* -308G>A (rs1800629)	forward primer	CCAAAAGAAATGGAGGCAATAGGTT
	reverse primer	GGACCCTGGAGGCTGAAC
	G probe	VIC-CCCGTCCCCATGCC-NFQ
	A probe	FAM-CCCGTCCTCATGCC-NFQ
*CCL2* -2518A>G (rs1024611)	forward primer	GGAGGGCATCTTTTCTTGACAGA
	reverse primer	GGAAGGTGAAGGGTATGAATCAGAA
	A probe	VIC-CAGACAGCTATCACTTT-NFQ
	G probe	FAM-AGACAGCTGTCACTTT-NFQ
*IL8* -251A>T (rs4073)	Assay-on-demand (C__11748116_10)	Predesigned assay from Applied Biosystems; primer and probe sequences not available.
*CCL5* -403G>A (rs2107538)	forward primer	ACTGAGTCTTCAAAGTTCCTGCTT
	reverse primer	GAGGACCCTCCTCAATAAAACACTTTATAAAT
	G probe	VIC-CATTACAGATCTTACCTCCTTT-NFQ
	A probe	FAM-CATTACAGATCTTATCTCCTTT-NFQ

Sequences of primers and probes for TaqMan genotyping assays are presented. FAM and VIC are names of fluorescent dyes. NFQ is the abbreviation for non-fluorescent quencher.

### Statistics

SPSS for windows (release 15.0.1; SPSS, Inc., Chicago, IL) was used for statistical analyses. Continuous variables were analyzed by Student’s *t*-test and presented as means±SD. Categorial variables were presented as percentages and were compared by the χ^2^ test. Odds ratios and 95% confidence intervals (CI) were calculated by logistic regression analysis, and genotypes were coded assuming an allele dose effect (wildtype genotype=0, heterozygous carrier of the mutated allele=1, homozygous carrier for the mutated allele=2). A p<0.05 was considered statistically significant.

## Results

Clinical characteristics of both groups are shown in Table 2. Arterial hypertension was significantly more prevalent in BRVO patients than among control subjects (76.9% versus 51.5%; p<0.001). Hypercholesterolemia and ever-smoking status were also higher among patients than in the control group; however neither characteristics were statistically significant (Table 2).

**Table 2:** Demographic data of patients with branch retinal vein occlusion and control subjects.

**Clinical characteristics**	**BRVO patients (n=398)**	**Control subjects (n=355)**	**p-value**
Females	228 (57.3%)	206 (58.0%)	0.84
Mean age (years±SD)	67.1±11.0	68.3±13.8	0.18
Arterial hypertension	306 (76.9%)	183 (51.5%)	<0.001
Diabetes mellitus	27 (6.8%)	24 (6.8%)	0.99
Hypercholesterolemia	310 (77.9%)	260 (73.2%)	0.14
Ever-smoker	119 (29.9%)	90 (25.4%)	0.16

Prevalence of arterial hypertension was significantly higher in branch retinal vein occlusion (BRVO) patients compared to control subjects. Data are mean±standard deviation, or number of subjects (%).

Table 3 presents genotype distributions and allele frequencies in BRVO patients and controls. Neither allele frequencies nor genotype distributions of any of the investigated gene polymorphisms were found to be significantly different between patients and controls. Observed genotype distributions were in line with those predicted by the Hardy–Weinberg equilibrium, and allele frequencies among controls were similar to those previously observed among other Caucasian populations [22,25,52,71-77]. In a logistic regression analysis, presence of BRVO was predicted by arterial hypertension, but not by any of the ten investigated gene polymorphisms (Table 4).

**Table 3:** Genotype and allele frequencies in patients with branch retinal vein occlusion and control subjects.

**Polymorphism**	**Genotype**	**BRVO patients**	**Control subjects**	**p-value**
*IL1B* -511C>T (rs16944)	CC	183 (46.0%)	162 (45.6%)	0.68
	CT	184 (46.2%)	159 (44.8%)	
	TT	31 (7.8%)	34 (9.6%)	
	T allele frequency	0.309	0.32	0.66
*IL1RN* 1018T>C (rs4251961)	TT	140 (35.2%)	145 (40.8%)	0.15
	TC	210 (52.8%)	162 (45.6%)	
	CC	48 (12.1%)	48 (13.5%)	
	C allele frequency	0.384	0.363	0.4
*IL4* -584C>T (rs2243250)	CC	287 (72.1%)	253 (71.3%)	0.58
	CT	106 (26.6%)	94 (26.5%)	
	TT	5 (1.3%)	8 (2.3%)	
	T allele frequency	0.146	0.155	0.62
*IL6* -174G>C (rs1800795)	GG	130 (32.7%)	115 (32.4%)	0.97
	GC	197 (49.5%)	174 (49.0%)	
	CC	71 (17.8%)	66 (18.6%)	
	C allele frequency	0.426	0.431	0.84
*IL10* -592C>A (rs1800872)	CC	216 (54.3%)	186 (52.4%)	0.14
	CA	161 (40.5%)	137 (38.6%)	
	AA	21 (5.3%)	32 (9.0%)	
	A allele frequency	0.255	0.283	0.22
*IL18* +183A>G (rs5744292)	AA	232 (58.3%)	204 (57.5%)	0.11
	AG	154 (38.7%)	129 (36.3%)	
	GG	12 (3.0%)	22 (6.2%)	
	G allele frequency	0.224	0.244	0.36
*TNF* -308G>A (rs1800629)	GG	277 (69.6%)	251 (70.7%)	0.95
	GA	115 (28.9%)	99 (27.9%)	
	AA	6 (1.5%)	5 (1.4%)	
	A allele frequency	0.16	0.154	0.75
*CCL2* -2518A>G (rs1024611)	AA	225 (56.5%)	206 (58.0%)	0.48
	AG	147 (36.9%)	133 (37.5%)	
	GG	26 (6.5%)	16 (4.5%)	
	G allele frequency	0.25	0.232	0.43
*IL8 *-251A>T (rs4073)	AA	76 (19.1%)	82 (23.1%)	0.23
	AT	215 (54.0%)	171 (48.2%)	
	TT	107 (26.9%)	102 (28.7%)	
	T allele frequency	0.539	0.528	0.68
*CCL5* -403G>A (rs2107538)	GG	272 (68.3%)	235 (66.2%)	0.63
	GA	113 (28.4%)	104 (29.3%)	
	AA	13 (3.3%)	16 (4.5%)	
	A allele frequency	0.175	0.192	0.4

Genotype and allele frequencies were not significantly different between branch retinal vein occlusion (BRVO) patients and control subjects, suggesting that these genotypes are not associated with BRVO risk.

**Table 4:** Logistic regression analysis of branch retinal vein occlusion risk.

**Risk factor**	**Odds ratio**	**95% Confidence Interval**	**p-value**
Arterial hypertension	3.33	2.42–4.57	<0.001
*IL1B* −511T	0.95	0.75–1.20	0.67
*IL1RN* 1018C	1.18	0.94–1.49	0.15
*IL4* −584T	0.87	0.64–1.18	0.37
*IL6* −174C	0.98	0.79–1.22	0.87
*IL10* −592A	0.83	0.65–1.05	0.12
*IL18* 183G	0.89	0.69–1.16	0.39
*TNF* −308A	1	0.74–1.36	0.98
*CCL2* −2518G	1.15	0.90–1.48	0.27
*IL8* −251T	1.06	0.85–1.31	0.61
*CCL5* −403A	0.86	0.66–1.13	0.28

Arterial hypertension, but none of the investigated gene polymorphisms, was significantly associated with branch retinal vein occlusion (BRVO) risk.

## Discussion

Increased rigidity of the retinal artery leading to compression of the underlying vein at an arteriovenous crossing site has strongly been implicated in the pathogenesis of BRVO [3]. We hypothesized that BRVO susceptibility might be conferred by functional gene polymorphisms that affected the expression of cytokines or chemokines, which themselves have all been shown to affect atherogenesis. In addition, some cytokines also exert procoagulatory effects and may thus affect BRVO risk by a second mechanism. To improve biologic plausibility gene polymorphisms were only selected as candidate risk factors when these polymorphisms had previously been associated with gene expression or activity of the gene product, and an essential role of the gene product in pro- or anti-inflammatory pathways had been known. Some of these polymorphisms have been associated with cardiovascular diseases, indicating that alterations in the genetics of the inflammatory system may modify the risk of these diseases [77-83].

Neither genotype distributions nor allele frequencies of the investigated gene polymorphisms differed significantly between BRVO patients and control subjects. The statistical power of the present study to detect a potential association between the investigated gene polymorphisms and BRVO was calculated for the polymorphism with the lowest minor allele frequency (*IL4−584C>T*). For this polymorphism, the present study had a statistical power of 0.80 to detect an odds ratio of ≥1.55, and a statistical power of 0.99 to detect an odds ratio of ≥2.0 for carriers of the mutated allele. For all other polymorphisms, the statistical power was equal or higher than that of the *IL4−584C>T* polymorphism. Thus, our results strongly suggest that none of these gene variants is likely a major risk factor for BRVO.

The expression of cytokines and chemokines is also affected by factors other than gene polymorphisms [26,49,57,62]. For example, the expression of MCP-1 can be induced by pro-inflammatory cytokines such as IL-1 and TFN-α [57]. Thus, our finding that the investigated gene polymorphisms were not associated with a significantly increased risk for BRVO does not necessarily argue against a role of these cytokines and chemokines in the pathogenesis of BRVO.

Large prospective studies are clearly warranted to elucidate whether altered plasma concentrations of both cytokines and chemokines are predictive for the development of BRVO. This, however, can only be achieved by conducting a prospective instead of a retrospective study. Yet, it is also possible that increased expression of inflammatory mediators may not be detected systemically in blood, but only locally in the vascular wall.

When interpreting the data of the present study, some limitations have to be considered. First, blood samples were obtained after the occurrence of BRVO. Thus, the present study is not suitable to investigate plasma cytokine and chemokine patterns. The aim of this study, however, was to investigate a hypothesized association between genetic variants and BRVO risk. As genotypes do not change during a person’s lifetime, the aforementioned limitation does not apply to the analysis of gene polymorphisms. Second, gene variants have been shown to vary between populations of different ethnic origins. Thus, our findings may not necessarily apply to ethnicities other than Caucasian. Finally, genetic studies are designed to compare allele or genotype frequencies between case and control groups. A statistical difference in frequencies between cases and controls suggests that the investigated genetic variant is associated with the disease. In the present candidate gene approach, we selected genetic polymorphisms on the basis of prior knowledge of gene function and allele frequencies. Thus, the candidate gene approach has the advantage of a higher probability of true association, which has been recognized as an important factor influencing the outcome of association studies [84]. The main limitation of the candidate gene approach is the usually low number of polymorphisms investigated. Whole genome association studies, including hundreds of thousands of polymorphisms, are now available. It is likely that results of such studies will provide novel candidates for genetic susceptibility to BRVO.

In conclusion, as none of the investigated gene variants was significantly more prevalent in BRVO patients than among control subjects, our data suggest that these polymorphisms are unlikely major risk factors for BRVO in a Caucasian population.
